# Effectiveness of an exercise intervention based on preactivation of the abdominal transverse muscle in patients with chronic nonspecific low back pain in primary care: a randomized control trial

**DOI:** 10.1186/s12875-023-02140-3

**Published:** 2023-09-06

**Authors:** Francesc Rubí-Carnacea, Maria Masbernat-Almenara, Carolina Climent-Sanz, Jorge Soler-González, María García-Escudero, Oriol Martínez-Navarro, Fran Valenzuela-Pascual

**Affiliations:** 1 Faculty of Nursing and Physiotherapy, Universidad de Lleida, Roig 2, 25198 Lleida, Montserrat, España; 2 Group of Studies on Society, Health, Education and Culture (GESEC), Universidad de Lleida, Pl. de Víctor Siurana 1, 25003 Lleida, España; 3 Research Group of Health Care (GReCS), Lleida Biomedical Research Institute’s Dr. Pifarré Foundation (IRB Lleida), Avda Alcalde Rovira Roure 80, 25198 Lleida, España; 4 Catalan Institute of Health, Rambla de Ferran 44, 25007 Lleida, España; 5 Faculty of Medicine and Health Sciences, Universidad Católica de Valencia San Vicente Mártir, Valencia, España

**Keywords:** Chronic nonspecific low back pain, Lumbopelvic stability, Motor control, Exercise, Transverse abdominal muscle

## Abstract

**Background:**

Low back pain is one of the most common disabling pathologies in humanity worldwide. Physical exercises have been used in recent decades to reduce the pain, improve the functionality of the lumbar spine and avoid relapses. The purpose of the study is to analyze the effect of a program based on re-education exercises involving preactivation of the abdominal transverse muscle compared to conventional treatment in adults with chronic nonspecific low back pain.

**Methods:**

A two-arm, single-blind randomized control trial with 35 primary care patients with chronic nonspecific low back pain. Both groups received a 4-week intervention. Data were collected at baseline and at the end of the intervention. Sixteen patients participated in the intervention group, and 19 patients in the control group.

**Results:**

For the experimental group, the outcomes of disability and activation of the abdominal transverse muscle decreased significantly (MD -2.9; CI 95% -5.6 to -0.35; η2 = 0.14; p = 0.028) and (MD 2.3; CI 95% 0.91 to 3.67; η2 = 0.25; p = 0.002) respectively, with a large effect size, compared to the control group. There were no differences between the groups in pain intensity, thickness, and resistance of the transverse abdominal muscle.

**Conclusion:**

A 4-week specific program based on re-education exercises of the preactivation of the abdominal transverse muscle is more effective than conventional treatment for reducing disability and increasing the activation of the abdominal transverse muscle measured by VAS scale and PBU.

**Trial registration:**

Clinicaltrials.gov identifier: NCT03097497. Date of registration: 31/03/2017.

**Supplementary Information:**

The online version contains supplementary material available at 10.1186/s12875-023-02140-3.

## Background

Low back pain (LBP) is one of the most common disabling pathologies worldwide [1]. In the last 30 years, in low- and middle-income Western societies, disability associated with LBP has become a massive problem due to the individual, health, work, economic, and social aspects that highlight the complexity of its causes and possible solutions [2]. Although most acute LBP usually resolves within four weeks, 2–15% of cases can become chronic [3].

In Western countries, the societal costs of this disease are estimated to be 1–2% of the gross national product, and between 80 and 90% of the costs are from productivity loss and disability [4]. In Spain, LBP is one of the most common reasons for visiting medical offices and physiotherapy, representing approximately 30% of medical visits [5].

LBP does not discriminate based on age, but the incidence increases as people grow older, peaking between 45 and 59 years, when it can become more disabling [3]. Focusing on the etiology, 80–85% of the cases are nonspecific, meaning there is no recognizable specific pathology [6]. These cases transition into chronic nonspecific low back pain (CNLBP), a high prevalence and low complexity entity that produces disability and work absenteeism [4].

CNLBP has been associated with lumbopelvic instability [7] and motor control dysfunction [8]. Some studies have shown that the improvement in dimensions and recruitment of deep muscles of the spinal column, including the transverse abdominal muscle (TrA), is related to improved function in the short term when patients with LBP conduct motor control exercises compared to general exercise [9].

The muscle’s preactivation system gives rise to anticipatory postural adjustments that position the body before the disturbances that occur during any movement [10]. These adjustments ensure proximal stability to allow distal mobility so that with the movement of the limbs (distal structure), the trunk musculature (proximal structure) activates first, thus preventing movement from destabilizing the spine [11].

## Methods

### Aim

This study aimed to analyze the effect of a program based on re-education exercises on the preactivation of the TrA muscle in terms of pain intensity, disability, activation measured with a pressure biofeedback unit (PBU), thickness measured with ultrasound (US), resistance measured with electromyography (EMG) compared with conventional treatment in adult people with CNLBP in primary care. We also investigated the relationship between the activation of the TrA muscle and pain and disability.

### Design

A longitudinal, single-blind, randomized controlled trial study was conducted between August 2017 and November 2018. This study was registered on clinicaltrials.gov (NCT03097497) on 31/03/2017 and followed the Consolidated Standards of Reporting Trials statement (CONSORT) recommendations to develop the structure and guide the performance of the study [12]. Subjects were recruited from different primary care centers in the city of Lleida. Data extraction was performed by an IDIAP Jordi Gol Research Unit technician following the inclusion/exclusion criteria.

The inclusion criteria were adults (18 to 65 years old) diagnosed with CNLBP, with a minimum of 3 months of follow-up.

The exclusion criteria were signs of neurological deficit, history of spinal surgery or cardiac disease, cancer or metastatic cancer treatment in the previous five years, and pregnancy or plan to become pregnant or less than three months postpartum.

### Sample size

The sample size calculation was based on the study by Unsgaard-Tøndel et al. [13]. It was assumed that the baseline pain in these patients would be approximately 3 ± 1.6 on a scale from 0 to 10. We assumed that a reduction in pain on the VAS scale of 2 points would be sufficient to consider the effectiveness of the treatment.

Accepting an alpha risk of 0.05 (95% confidence) and a beta risk of 0.1 (statistical power of 90%), using a two-sided contrast, 17 individuals were necessary in each group (34 patients in total), accepting a patient drop-out rate of 20%. We used the sample-size calculator GRANMO version 7.12.

A simple random sampling method without replacement was used to ensure a representative sample of our target population.

### Randomization and blinding

The researchers used a simple randomization technique. An external researcher generated the randomization assignment using a computer random number generator in Excel 2011 (version 14.0.0) and kept the assignments on a specific computer for this study. The group assignments were inaccessible to the rest of the staff. Neither the participants nor the investigators responsible for enrolling the patients could foresee the assignment because of the central allocation used for this study. To ensure that the assessment of the patients was not biased, we used an external assessor who was blinded to their group assignment.

### Interventions

The intervention was carried out at the Faculty of Nursing and Physiotherapy of the University of Lleida, Spain. Based on several previous studies, the duration of the intervention was four weeks [14]. Two physiotherapists were instructed in the intervention and had to pass a qualification test.

The patients assigned to the control group followed the conventional treatment prescribed by their family physician during the primary care consultation, following the guidelines of the Catalan Health Institute [15]. The most recommended treatment consisted of education regarding lumbar symptoms, recommendations to stay active, and the use of medications such as paracetamol and NSAIDs (Non-Steroidal Anti-Inflammatory Drugs).

Patients assigned to the experimental group followed a program of re-education exercises for preactivation of the transverse abdominal muscle for approximately 30 min each session. The sessions were conducted individually to ensure adequate attention and individualization of the exercises. Each intervention session was divided into warm-up and TrA muscle training (supplementary file).

Individualized parameters were established for each patient to obtain optimal results in motor control. This individualized training method has been described in several articles where a PBU has been used as an element of assessment and feedback [16]. PBU is an instrument that consists of a nonelastic air bladder that detects the pressure fluctuations implicit in movements in that area when placed between the supporting surface and the lumbar spine [8].

By transferring this procedure to the lumbar muscles combined with the optimal resistance training parameters described by Borde et al. [17], the maximum capacity that the patient can perform with an abdominal contraction can be measured with the PBU (mmHg) by calculating 70–79% of the maximum repetition (1RM). The initial resistance of each patient was defined as 70–79%, meaning the number of seconds that the patient can hold an abdominal contraction, up to a maximum of 6 s. The number of repetitions the patient can perform is assessed at up to 9 repetitions. The patient performed a maximum of 3 series, optimizing the capacity of each patient and avoiding fatigue.

To facilitate the contraction of the deep trunk muscles, the PBU was used to give feedback information, and they were ordered to perform the drawing-in maneuver (bring the abdomen in and up). According to Richardson et al. [18], this feedback helps ensure precision in the exercise and guides the progression.

By abdominal palpation and observation, the physiotherapist verified whether the patients performed this action correctly, without compensation by pelvic retroversion, trunk rotations, or the contraction of any nearby muscles, if no verbal feedback was given to improve the movement. Furthermore, to facilitate the action of this abdominal musculature, the patient was asked to contract the pelvic floor musculature. In this case, the order given was ‘squeeze your ass’, ‘put your ass in’, and ‘hold your urine’.

Verbal and tactile reinforcement was given to the patient to hold the contraction for 6 s. If the patient did not achieve that, we worked with the time that the patient was able to hold it. After the contraction, the patient rested for four seconds, and then the contraction was repeated to see how many repetitions the patient could perform. Then, the patient rested for 60 s before performing another series until a maximum of three and a minimum of two series for each exercise. Following these parameters, four different exercises were performed in each session, progressively increasing the weekly training load and difficulty.

The exercises are available in a supplementary file.

#### Outcomes

The intervention lasted for four weeks. All variables were measured pre- and post-test. For all of the variables, whenever possible, the minimal clinically important difference (MCID) to be detected was established. The study’s main variable was pain intensity measured with a VAS scale of 0–10 cm [19]. The MCID for the VAS was established as 2 points [20]. The secondary variables were disability and the ability to activate the transverse abdominal muscle. Disability was measured with the Roland-Morris Questionnaire (RMQ) [21]. For the RMQ, a change greater than 4 points was considered the MCID [22]. PBU, EMGs, and US were used to better measure the transverse abdominal muscle’s activation capacity.

**Fig. 1:**
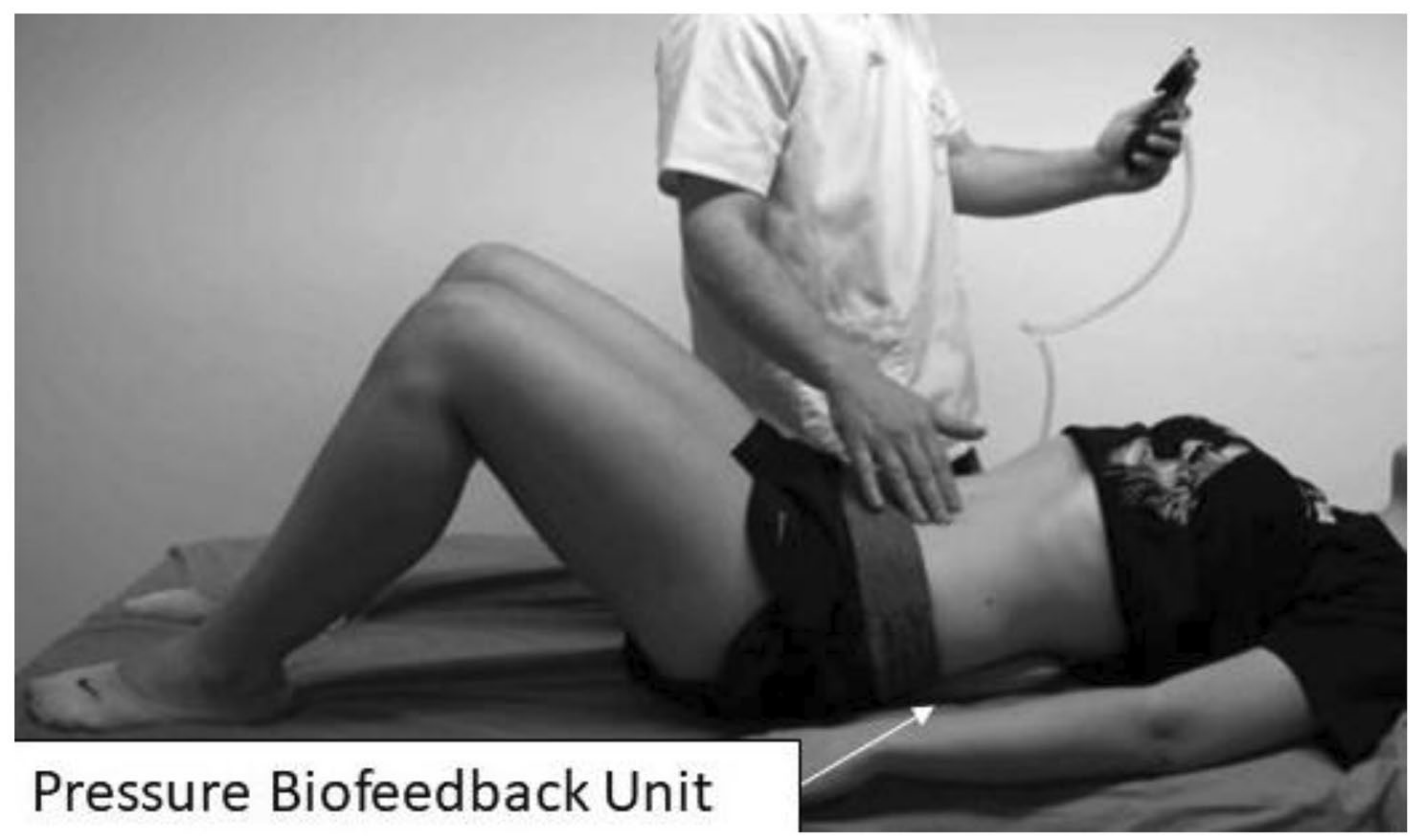
Pressure biofeedback unit

The PBU was inserted under the subject’s lumbar spine, between the ribcage and the sacrum, in the area corresponding to the thoracolumbar fascia. It was inflated to an initial pressure of 40 mmHg. The patient was supine on a stretcher, with the knees flexed at 90º and a pillow under the neck to maintain a comfortable and neutral posture (Fig. 1).

Before the test, all the participants were instructed to perform the abdominal contraction maneuver to focus the action on the transverse abdominis, not the rectus or obliques abdominis. Several contraction trials were allowed before the recording was taken until the performance was judged optimal by the physiotherapist. A prudential rest time of 2 min was allowed so that fatigue did not intervene in the assessment result, with the minimum being 30–60 s [17, 23].

The drawing-in abdominal contraction maneuver was requested to record the test result, and the peak pressure change was taken into account with a record of 10 s of contraction. The result was calculated by identifying the peak point of contraction held for more than 1 s and then subtracted from the baseline pressure. Three trials were used for statistical analyses, taking the average of the three contractions.

The bioPlux surface electromyograph was used with its corresponding software and TIGA-MED gold bipolar surface electrodes.

Before placing the electrodes, the detection surfaces were adequately cleaned and shaved when necessary, following the recommendations of the ISEK (International Society of Electrophysiology and Kinesiology) and SENIAM (Surface Electromyography for Non-Invasive Evaluation of Muscles) to allow a low impedance between the skin and the electrodes [24, 25].

First, the reference electrode or ground electrode was placed on the ankle, above the right external malleolus, since it is one of the places with the least electrical activity in the body. This electrode was connected to the G channel of the bioPlux device and waited for 30 s. The reference electrode collects the basal electrical signal, which is always present on the skin surface and is not the result of muscle activity. Next, the two electrodes of channel 1 were placed on the area corresponding to the TrA/OI (obliquus internus abdominis) muscles, located 2 cm from the anterior superior iliac spine and with a distance of 2 cm between their centers [24–26].

The center and edges of the electrode detection zones were pressed firmly to ensure good contact with the patient’s skin. Initially, the patient’s basal tone was measured for 30 s. Once the result was recorded, the maximal voluntary isometric contraction of the TrA was requested following the SENIAM standards to obtain the maximum force peak. Three contractions of 5 s each with 3 s of rest between repetitions were requested. Two more repetitions of the entire sequence were performed with a 60-second rest between each sequence. By recording a very low-intensity signal mixed with other undesirable ones, the signal was amplified x1000, and the bandpass was filtered from 20 to 450 Hz, quantifying it afterwards. All these results were recorded, and the bioPlux device calculated the average, giving the mV (millivolts) values of the force test as a result.

Finally, 10 s of maximum contraction of the TrA were requested to assess muscle resistance. This contraction was also recorded due to the resistance test with mV.

For the measurement of TrA, we used the bioPLUX wireless System Surface US. The patient was placed in a quadruped position. The conductive gel was applied to a transducer at 7.5 MHz. This was placed transversely on the right side of the body, with the center positioned at a point 2.5 cm anterior to the axillary midline, at the midpoint between the last rib and the iliac crest. Once a clear image of the TrA was obtained, it was measured at rest, freezing the image at the end of the patient’s exhalation and measuring the width at its widest point. The patient was then asked to perform an abdominal maximal voluntary isometric contraction, and the image was re-frozen at the end of the patient’s expiration for measurement. A total of three images of the contracting TrA were captured, and muscle thickness measurements in millimeters were averaged.

### Statistical analysis

The statistical analysis was performed using the statistical program SPSS v22, and the intention-to-treat analysis was performed with an alpha of 0.05. Quantitative variables were described using the mean, standard deviation (SD), standard error (SE), median, and interquartile range.

Sociodemographic baseline characteristics were compared using the chi-square tests of independence for categorical data and the Student’s t-test for continuous data.

A two-way mixed ANOVA was used to determine whether there was an interaction effect between the two independent variables, treatment (control and experimental) and time (pre- and post-test). The mean difference (MD) with 95% confidence interval (CI) was calculated to analyze continuous outcomes. We corrected p-value comparisons using Bonferroni. We used the partial Eta squared (η2) to measure the effect size. We considered a partial η2 > 0.009 as a small effect size, partial η2 > 0.058 as a medium effect size, and partial η2 > 0.137 as a large effect size [27, 28].

We used the Pearson bivariate correlation analysis to investigate the relationship between the TrA activation measured with the PBU and pain and disability.

## Results

**Fig. 2:**
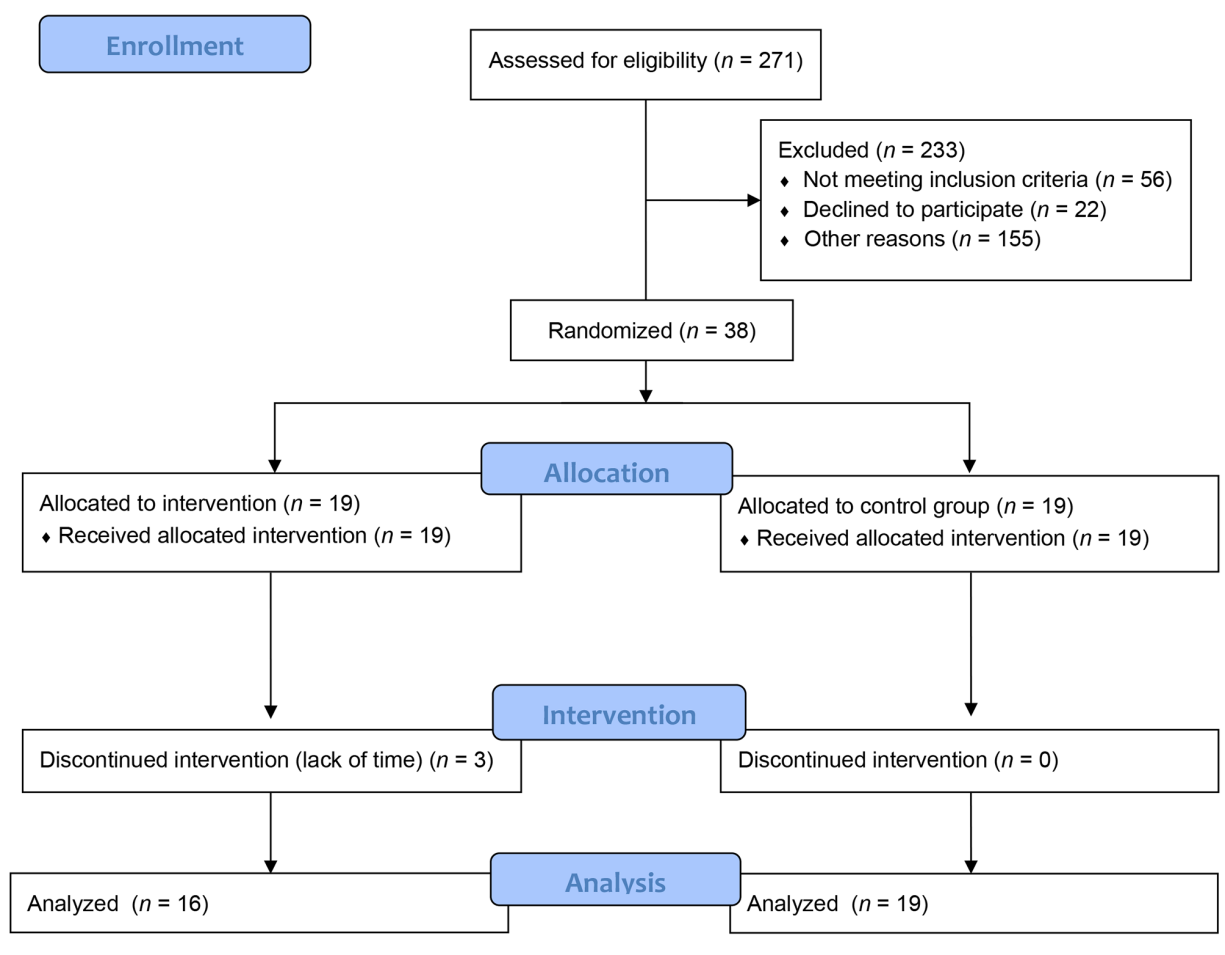
CONSORT Flow diagram of the study

Recruitment took place in different primary care centers of the “Institut Català de la Salut” in Lleida (Spain) from April to August 2017. Of 271 potential subjects, 155 were impossible to contact, and 78 were excluded after the telephone interview. Figure 2 shows the flow chart of the study.

Finally, 38 patients participated in the study (see Table 1). The participants were randomized into the control group (n = 19) or the intervention group (n = 19). Of the 38 patients who started the study, 92.1% finished and completed the pre- and post-treatment assessments. Only three patients from the intervention group dropped out of the study because of the lack of availability to attend twice a week to carry out the treatment. Due to the higher degree of involvement of the subjects in the intervention group compared to the control group, it was more likely that drop-outs occurred in this group.

The initial analysis of the groups showed no significant difference in the quantitative demographic characteristics, such as age, BMI, or the onset of the first episode of LBP. For the qualitative variable sex, it was found that in the control group, 61% were men compared to 39% women, while in the intervention group, the percentage of men was only 29%, but this difference was not significant according to Pearson’s chi-square test (p = 0.23) (Table 1).

**Table 1:** Baseline Characteristic of participants

	Total (n = 35)	Control (n = 19)	Intervention (n = 16)	Control vs. Intervention *(P)
Sex	18 W, 17 M	8 W, 11 M	10 W, 6 M	0.229
Age (years)	43.5 (6.7)	43.3 (4.3)	43.8 (8.8)	0.486
Body Mass Index (kg/m2)	24.8 (3.7)	25.9 (4)	23.5 (3)	0.069
First episode LBP (years)	11.1 (7.4)	9.9 (5.6)	12.5 (9.1)	0.517
VAS (0–10)	5 (2.5)	4.6 (2.7)	5.4 (2.3)	0.529
RMQ (0–24)	7.5 (4.8)	6.8 (4.9)	8.3 (4.7)	0.238
Activation TrA - PBU (mmHg)	2.3 (2.25)	2.7 (2.71)	1.9 (1.5)	0.641
Electric Activity TrA - EMGs				
*Resistance 10s (mV)*	0.053 (0.06)	0.045 (0.03)	0.064 (0.08)	0.75
Thickness TrA - US				
*CSA resting (mm)*	3.76 (1.61)	3.87 (1.77)	3.63 (1.45)	0.728
*CSA contraction (mm)*	6.19 (1.99)	6.51 (2.33)	5.81 (1.49)	0.436
*CSA improvement (mm)*	2.42 (1.25)	2.63 (1.24)	2.18 (1.25)	0.389

Notes: Values are presented as mean (standard deviation)

Abbreviations: W women; M Men; LBP Low Back Pain; TrA; abdominal transversus muscle; RMQ Roland-Morris Questionnaire; PBU Pressure Biofeedback Unit; US Ultrasound; CSA: cross-sectional area

### Main outcome

The main outcome in this study was pain intensity measured with a 0–10 VAS scale. The measurements were taken pre- and post-test before the start of the intervention and after four weeks (Tables 2 and 3, and Table 4).

**Table 2:** Results experimental group

Experimental group (n = 16)			
**Outcome**	**Pre** Mean (SD)	**Post** Mean (SD)	**p value**
VAS (0–10)	5.4 (2.3)	3.3 (2.5)	0.00*
RMQ (0–24)	8.3 (4.7)	4.5 (2.9)	0.00*
Activation TrA-PBU (mmHg)	1.9 (1.5)	4.4 (1.44)	0.00*
Thickness TrA-US (mm)	2.18 (1.25)	2.91 (1.34)	0.01*
Resistance TrA-EMG (mV)	0.064 (0.081)	0.073 (0.08)	0.33

*Statistically significant difference. SD: Standard Deviation VAS: Visual Analogue Scale. RMQ: Roland-Morris Questionnaire. TrA: Abdominal Transverse Muscle. PBU: Pressure Biofeedback Unit. US: Ultrasound. EMG: Electromyography

**Table 3:** Results control group

Control group (n = 19)			
**Outcome**	**Pre** Mean (SD)	**Post** Mean (SD)	**p value**
VAS (0–10)	4.6 (2.7)	4.5 (2.6)	0.71
RMQ (0–24)	6.8 (4.9)	7.5 (4.4)	0.43
Activation TrA-PBU (mmHg)	2.7 (2.7)	2.1 (2.3)	0.18
Thickness TrA-US (mm)	2.63 (1.24)	2.41 (1.28)	0.24
Resistance TrA-EMG (mV)	0.045 (0.03)	0.044 (0.034)	0.95

*Statistically significant difference. SD: Standard Deviation VAS: Visual Analogue Scale. RMQ: Roland-Morris Questionnaire. TrA: Abdominal Transverse Muscle. PBU: Pressure Biofeedback Unit. US: Ultrasound. EMG: Electromyography

A two-way mixed ANOVA was conducted to examine the effects of time and treatment on pain intensity. The interaction effect between time and treatment on pain intensity was statistically significant (F(1, 33) = 14.33, p = 0.001, η2 = 0.3). There was a significant main effect of the intervention (differences between the measurements pre- and post-intervention) on pain intensity (F(1, 33) = 18.4, p = 0.00, η2 = 0.36). For the experimental group, pain intensity was significantly decreased post-test compared to pre-test (MD -2.12; CI 95% -2.9 to -1.33; p = 0.00) (Table 2). The main effect for treatment was not statistically significant (p = 0.17) (Table 4).

**Table 4:** Comparative results

Results (n = 35)					
**Outcome**	**Group**	**Mean change** (SE)	**Mean difference** **(95% CI)**	**Effect size**	***p*** **value**
VAS (0–10)	Control (19) Experimental (16)	-2.1 (0.38) -0.1 (0.35)	-1.2 (-2.98 to 0.56)	0.05	0.17
RMQ (0–24)	Control (19) Experimental (16)	0.7 (0.8) -3.8 (0.87)	-2.9 (-5.6 to -0.35)	0.14	0.02*
Activation TrA-PBU (mmHg)	Control (19) Experimental (16)	-0.6 (0.37) 2.5 (0.4)	2.3 (0.91 to 3.67)	0.25	0.02*
Thickness TrA-US (mm)	Control (19) Experimental (16)	-0.22 (0.1) 0.74 (0.2)	0.5 (-0.4 to 1.4)	0.03	0.26
Resistance TrA-EMG (mV)	Control (19) Experimental (16)	-0.001 (0.009) 0.009 (0.01)	0.03 (-0.01 to 0.07)	0.06	0.15

*Statistically significant difference. SE: Standard Error. CI: Confidence Interval. VAS: Visual Analogue Scale. RMQ: Roland-Morris Questionnaire. TrA: Abdominal Transverse Muscle. PBU: Pressure Biofeedback Unit. US: Ultrasound. EMG: Electromyography. Partial Eta squared, F and p-values were calculated using a two-way mixed ANOVA

### Secondary outcomes

There were four secondary outcomes: (i) disability measured with the RMQ, (ii) activation of the transverse abdominal muscle using the pressure biofeedback unit, (iii) abdominal transverse muscle thickness measured with ultrasound, and (iv) resistance of the transverse abdominal muscle measured with electromyography. A two-way mixed ANOVA was conducted to examine the effects of time and treatment on all secondary outcomes.

#### Disability (RMQ)

The interaction effect between time and treatment on disability was statistically significant (F(1, 33) = 13.64, p < 0.001, η2 = 0.3). There was a significant main effect of the intervention (differences between measurements pre- and post-intervention) on disability (F(1, 33) = 6.9, p = 0.013, η2 = 0.17). For the experimental group, disability was significantly decreased compared to the control group (MD -2.9; IC 95% -5.6 a -0.35; η2 = 0.14; p = 0.028), with a large effect size (Table 4). For the experimental group, disability was significantly decreased post-test compared to pre-test (MD -3.75; IC 95% -5.52 to -1.97; p = 0.00) (Table 2).

#### Activation of the Abdominal Transverse Muscle (PBU)

The interaction effect between time and treatment on pain intensity was statistically significant (F(1, 33) = 32.59, p = 0.00, η2 = 0.49). There was a significant main effect of the intervention (differences between measurements pre- and post-intervention) on the activation of the transverse abdominal muscle using the pressure biofeedback unit (F(1, 33) = 12.52, p = 0.001, η2 = 0.27). For the experimental group, the activation of the transverse abdominal muscle using the pressure biofeedback unit was significantly increased compared to that of the control group (MD 2.3; CI 95% 0.91 to 3.67; η2 = 0.25; p = 0.002), with a large effect size (Table 4). For the experimental group, the activation of the transverse abdominal muscle using the pressure biofeedback unit was significantly increased post-test compared to pre-test (MD 2.54; CI 95% 1.72 to 3.36; p = 0.00) (Table 2).

#### Abdominal Transverse Muscle Thickness (US)

The interaction effect between time and treatment on abdominal transverse muscle thickness was statistically significant (F(1, 33) = 12.12, p = 0.001, η2 = 0.27). For the experimental group, the abdominal transverse muscle thickness measured with US was significantly increased post-test compared to pre-test (MD 0.74; CI 95% 0.03 to 0.11; p = 0.001) (Table 2). There were no statistically significant differences between groups (Table 4).

#### Resistance of the Abdominal Transverse Muscle (EMG)

The interaction effect between time and treatment on the resistance of the transverse abdominal muscle was not statistically significant (F(1, 33) = 0.58, p = 0.45, η2 = 0.17). There were no statistically significant differences within and between groups (Table 4).

#### Correlation analysis between the Activation of the Abdominal Transverse Muscle (PBU) and pain (VAS), disability (RMQ) and Abdominal Transverse Muscle Thickness (US)

A statistically significant negative correlation was found between TrA activation and pain (r= -0.358, p = 0.035).

There was no correlation between TrA activation and disability.

A statistically significant correlation was found between TrA activation and TrA thickness (r = 0.474, p = 0.004).

## Discussion

Our findings suggest that a specific program based on re-education exercises on the preactivation of the transverse abdominal muscle significantly reduces disability and increases the activation capacity of the transverse abdominal muscle in the short term compared to a conventional treatment that includes education about lumbar symptoms, recommendations to be active and pharmacological prescriptions, in adults with CNLBP.

Our results showed no statistically significant differences between the two groups for pain intensity measured with the VAS. This result is in contrast with the literature. A recent systematic review with meta-analysis and meta-regression showed low to moderate quality evidence of a sustainable positive effect of motor control exercise on pain intensity [29]. This difference in results may have been due to the duration of the intervention. While the intervention in our study lasted four weeks, the mean duration of the intervention in the ten studies included in the review was eight weeks [29]. This 4-week difference in the duration of the intervention may have been relevant in significantly reducing pain intensity in the experimental group compared to the control group.

The experimental group had significantly decreased disability measured with the RMQ scale compared to the control group. These results are consistent with a meta-analysis [30], which affirms that a specific lumbar stabilization program is better than general treatments in reducing disability in patients with CNLBP. Other reviews [31] also concluded that stabilization and motor control exercises significantly reduced disability. However, these reviews cannot prove that the proposed exercises are better than other general treatments [31]. A recent study with 70 patients diagnosed with low back pain underwent education and low-load motor control exercises compared to education and high-load lifting exercises. The study showed a significant improvement in terms of disability in the group that performed low-load motor control exercises. In another study, the authors concluded that the group that performed specific stabilization exercises had significantly reduced disability compared to the group that performed only McKenzie exercises [32]. According to our results, a specific lumbar stabilization treatment would always obtain more benefits concerning disability than conventional treatments without a specific program, including recommendations of being active and education about symptomatology to be relieved through pharmacological prescriptions.

Regarding the activation of the transverse abdominal muscle using the PBU, our results showed a statistically significant difference in favor of the experimental group. Several studies [8] have shown that the PBU is not a valid tool to measure abdominal transverse muscle activation in people with CNLBP. However, there is evidence that confirms the usefulness of PBU for biofeedback purposes to increase the activity of the abdominal muscles in people with LBP [8]. In line with our results, other types of exercise, such as equipment based and mat Pilates, have been proven effective in improving the activation of the transverse abdominal muscle [1].

Comparing the results obtained from the PBU and the US regarding the activation of the TrA, both measures showed a significant correlation. However, although the results obtained with the PBU are significantly increased in favor of the experimental group, the results of the US are not statistically significant. As mentioned, some evidence shows that the PBU is not a valid tool for measuring TrA activation [8]. In a systematic review, de Paula Lima et al. [33] concluded that “The current evidence about the measurement properties of PBUs for the assessment of TrA activity is mainly based on studies with suboptimal designs, and the findings from these studies are likely to be overly optimistic.”

Finally, there were no statistically significant differences between the two groups for the thickness and resistance of the transverse abdominal muscle as measured by US and EMG, respectively. These results are in contrast with the literature. In a similar study [32], the authors concluded that patients who performed lumbar stabilization exercises significantly increased the thickness of the transverse abdominal muscle compared to patients who only performed Mckenzie exercises. Another study compared specific lumbar stabilization exercises with abdominal training using the “drawing” technique guided with PBU [34]. That study concluded that in both groups, there was an improvement in muscle thickness; however, specific lumbar stabilization exercises have more benefits in terms of spinal stability [34]. According to the literature, there is not enough evidence regarding the reliability of measurements of the transverse abdominal muscle using US due to the intraobserver variability and the variability in the measurement protocols [35]. However, some studies have confirmed the effectiveness of using US as feedback to optimize transverse abdominal muscle activation during exercise [36].

The literature generally establishes that greater activation of the transverse abdominal muscle occurs when we work with suspension training systems or perform CORE stabilizing exercises [37]. Our study obtained results similar to a previous study [38], which concluded that the activity of the transverse abdominal muscle measured by EMG did not show a significant individual variation. However, the same authors determined that changes in the thickness of the transverse abdominal muscle may indicate changes in the electrical activity of this muscle [38].

Finally, in our study, we have conducted an intention to treat analysis. This method analyzes patients according to the groups they were initially assigned and randomized. For some authors, this method preserves the prognostic balance that randomization offers [39]. Interestingly, a systematic review by Matheve et al. [40] on technology-supported exercise therapy for LBP showed that from the 25 studies reviewed, only nine studies analyzed the results using the intention to treat method. As asserted by Detry and Lewis [41], “Only by retaining all patients intended to receive a given treatment in their original treatment group can researchers and clinicians obtain an unbiased estimate of the effect of selecting one treatment over another.”

### Limitations

The main limitation of this study is the placement of the US. The professionals were adequately instructed in the use of it and where they must place it to perform the measurement, but due to the morphological variability of each patient, the US position could have been slightly modified, which could cause intraobserver variations [36]. Another limitation of this study is that only the short-term effect was studied; we could not estimate the intervention’s effect in the medium or long term as we did not conduct a follow-up.

## Conclusions

The main conclusion is that a 4-week specific program based on re-education exercises on the preactivation of the transverse abdominal muscle is more effective than conventional treatment for reducing disability and increasing the activation of the transverse abdominal muscle measured by the RMQ and PBU.

Additional studies are necessary to estimate this treatment’s effect in the medium and long term. This program should also be compared with other exercise programs with supporting evidence, such as Pilates or fit-ball exercises.

### Electronic supplementary material

Below is the link to the electronic supplementary material.

Supplementary Material 1

## Data Availability

The datasets generated during the current study are available from the first author on reasonable request.

## Abbreviations

CNLBP: Chronic Nonspecific Low Back Pain
EMG: Electromyography
LBP: Low back pain
PBU: Pressure Biofeedback Unit
RM: Maximum Repetition
RMQ: Roland-Morris Questionnaire
TrA: Abdominal transversus muscle
VAS: Visual Analogic Scale
