# Supplementary material for: Effectiveness of an exercise intervention based on preactivation of the abdominal transverse muscle in patients with chronic nonspecific low back pain in primary care: a randomized control trial
Source: BMC Prim Care. 2023 Sep 6;24:180. doi: 10.1186/s12875-023-02140-3 (PMC10483714; doi:10.1186/s12875-023-02140-3)
Supplement: Supplementary file 1 — Supplementary Material 1 [file 12875_2023_2140_MOESM1_ESM.doc]

Training sessions

| **Week** | **Specific exercise** | |
| --- | --- | --- |
| Week 1 | Warm-up A. Supine position | 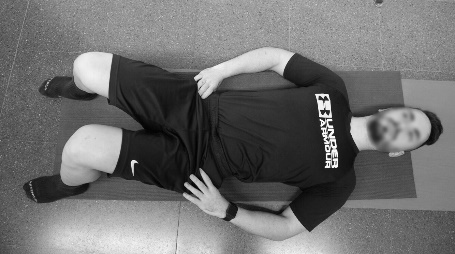 |
|  | 1. Basic prone position | 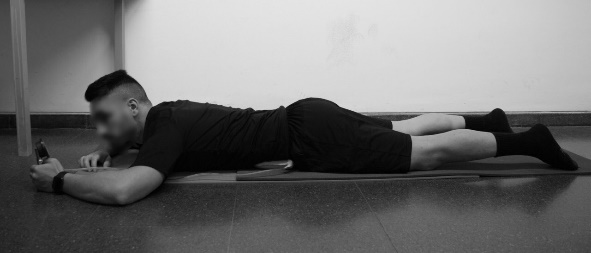 |
|  | 2. Basic supine position | 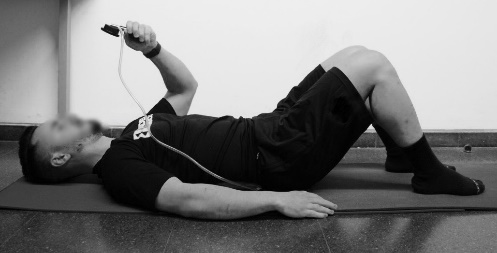 |
|  | 3. Basic sitting | 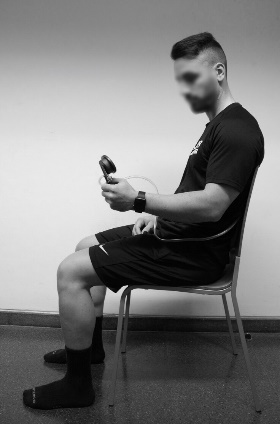 |
|  | 4. Basic standing | 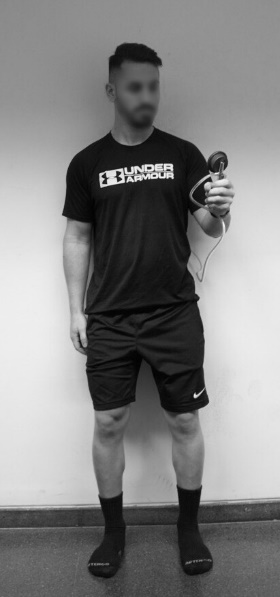 |
| Week 2 | Warm-up B. Standing with wall support | 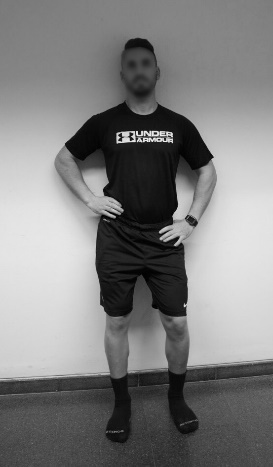 |
|  | 5. Prone position with upper limbs (UL) and lower limbs (LL) movement | 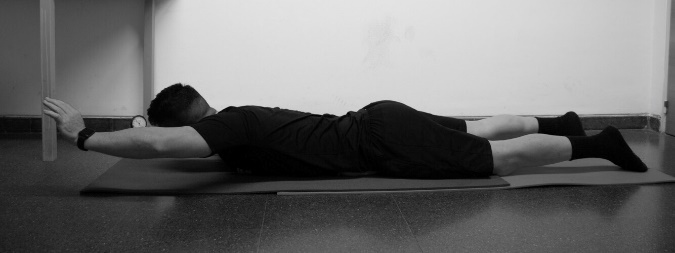 |
|  | 6. Supine position with UL and LL movement | 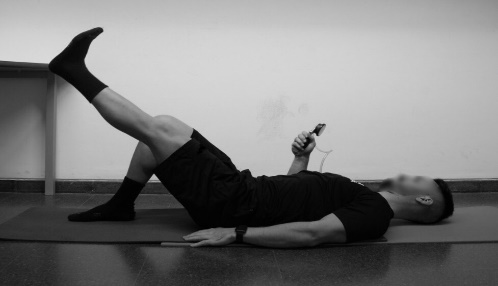 |
|  | 7. Sitting with UL and LL movement | 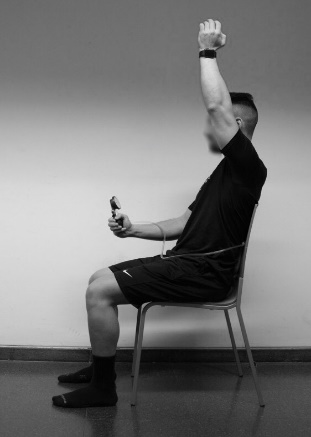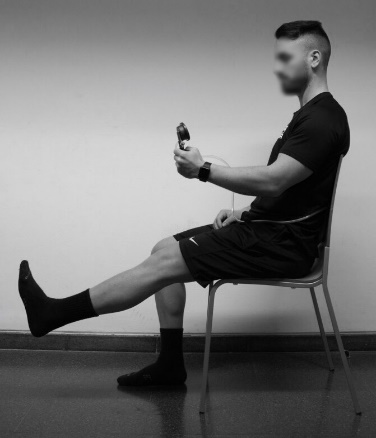 |
|  | 8. Standing with forearm support | 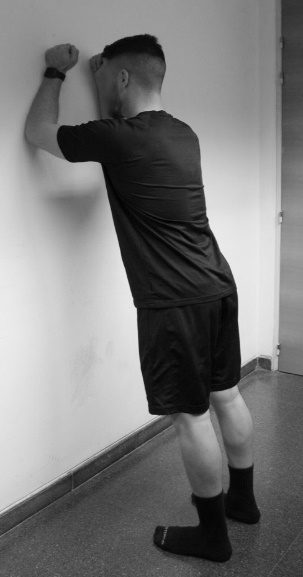 |
| Week 3 | Warm-up C. Sitting | 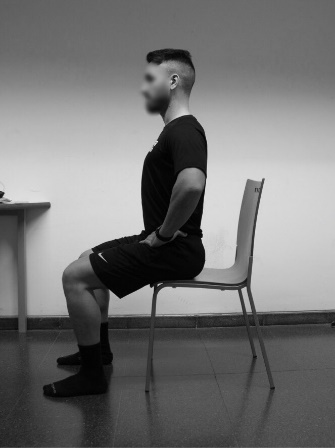 |
|  | 9. Quadruped with UL and LL movement | 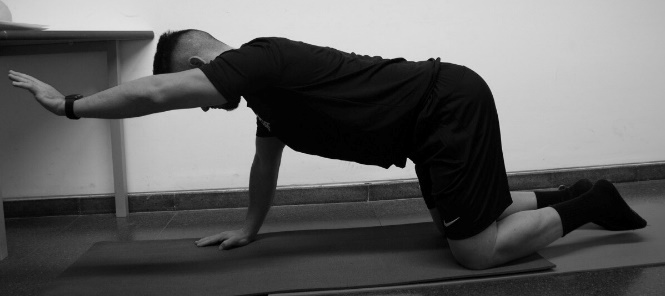 |
|  | 10. Supine with upper roll | 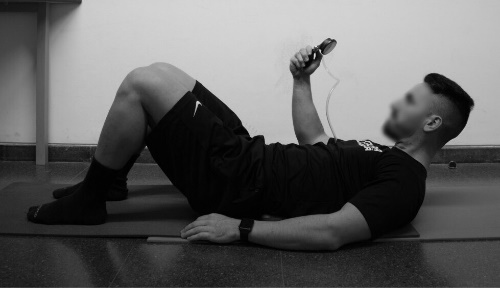 |
|  | 11. Sitting with instability with UL and LL movement | 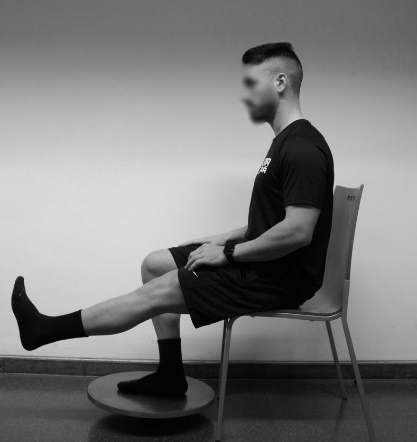 |
|  | 12. Lateral standing | 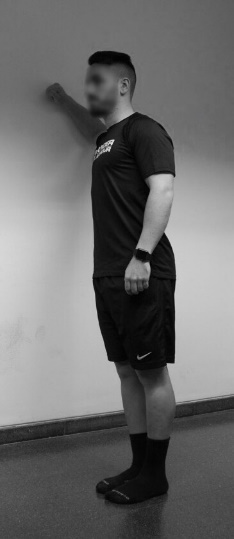 |
| Week 4 | Warm-up D. Standing without support | 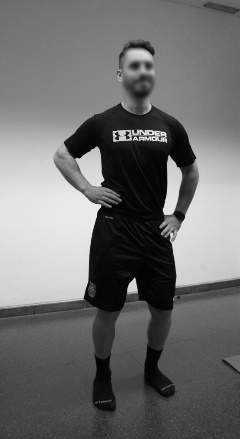 |
|  | 13. Unstable prone position | 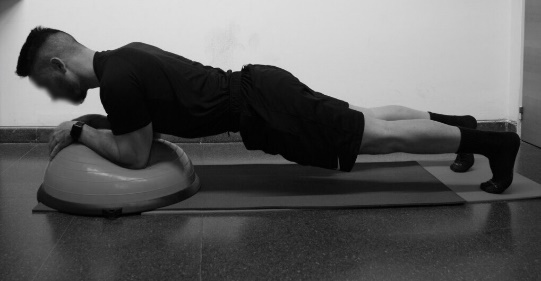 |
|  | 14. Supine with lower roll | 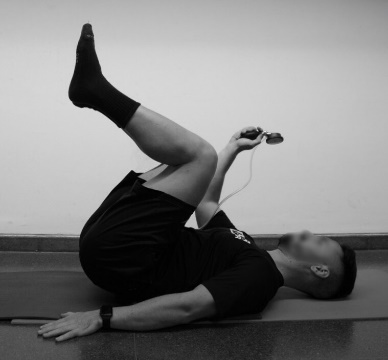 |
|  | 15. Unstable sitting with UL and LL movement | 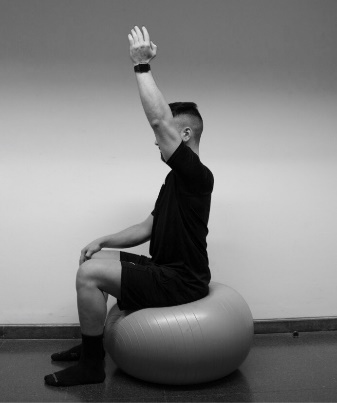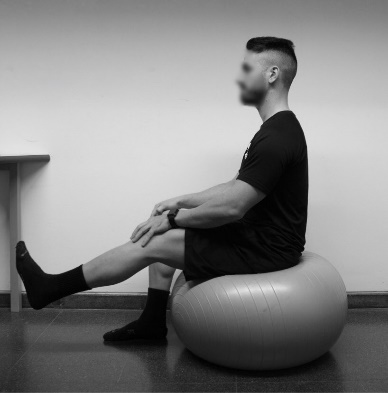 |
|  | 16. Unstable lateral standing | 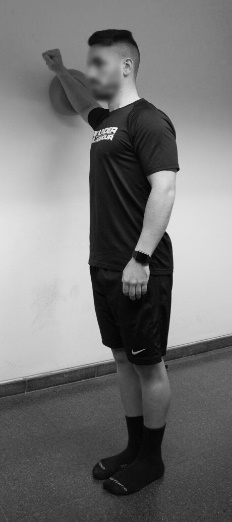 |
